# Impact of histologic subtypes and treatment modality among patients with primary central nervous system lymphoma: a SEER database analysis

**DOI:** 10.18632/oncotarget.25622

**Published:** 2018-06-22

**Authors:** Dai Chihara, Nathan H. Fowler, Yasuhiro Oki, Michelle A. Fanale, Loretta J. Nastoupil, Jason R. Westin, Luis E. Fayad, Sattva S. Neelapu, Chan Yoon Cheah

**Affiliations:** ^1^ Department of Internal Medicine, University of New Mexico, Albuquerque, NM, USA; ^2^ Department of Lymphoma/Myeloma, The University of Texas MD Anderson Cancer Center, Houston, TX, USA; ^3^ Department of Haematology, Sir Charles Gairdner Hospital, Nedlands, Western Australia, Australia; ^4^ Department of Haematology, Pathwest Laboratory Medicine WA, Nedlands, WA, Australia; ^5^ Medical School, University of Western Australia, Crawley, WA, Australia

**Keywords:** primary central nervous system lymphoma, SEER, marginal zone lymphoma, follicular lymphoma, survival

## Abstract

Primary central nervous system lymphoma (PCNSL) is a rare and aggressive extranodal presentation of lymphoma; however, the data for outcomes of patients with subtypes other than diffuse large B-cell lymphoma (DLBCL) are limited. Therefore, we analyzed overall survival (OS) of adult patients diagnosed with PCNSL by histologic subtype between 1998 and 2014 using the Surveillance, Epidemiology and End Results. A total of 4375 patients were identified. The median age of the patients was 64 years (range: 18-96). DLBCL was the most common histology (N=3,091), followed by follicular lymphoma (FL, N=83), peripheral T-cell lymphoma (PTCL, N=64), marginal zone lymphoma (MZL, N=63), Burkitt lymphoma (BL, N=27), small lymphocytic lymphoma (SLL, N=22), Hodgkin lymphoma (HL, N=13) and others (N=1,012). The 5-year OS rates were 30% in DLBCL, 66% in FL, 33% in PTCL, 79% in MZL, 42% in BL, 38% in SLL and 45% in HL. Radiation alone showed similar OS compared to no treatment in DLBCL, BL and PTCL, while radiation alone was associated with similar OS to chemotherapy or chemo-radiation in FL and MZL. The outcomes of patients with PCNSL are unfavorable; with the exception of FL and MZL which can potentially show prolonged survival with surgical resection or radiation monotherapy.

## INTRODUCTION

Primary central nervous system lymphoma (PCNSL) is a rare extranodal presentation that represents 1-2% of all non-Hodgkin lymphoma (NHL), however recent data indicate increasing incidence, particularly among the elderly [1, 2].

PCNSL is typically associated with an aggressive clinical course and poor outcome; however, there has been recent progress in treatment strategies. Current National Comprehensive Cancer Network (NCCN) guidelines for PCNSL recommend HD-MTX based induction regimen or whole brain radiation (WBRT) for patients who are not candidates for systemic chemotherapy [3]. The introduction of HD-MTX based regimen in the last two decades has resulted in substantial improvements in survival. Multiple trials have showed significant improvement in survival using HD-MTX based regimen compared to WBRT alone with a median OS of 30 to 60 months [4–8]. DeAngelis et al first showed that adding HD-MTX and high-dose cytarabine to WBRT significantly improved PFS compared to WBRT alone (median PFS: 10 months vs 41 month) [8], later confirmed in a multicenter study [5]. Although HD-MTX was initially introduced with WBRT, it became apparent among long-term survivors that delayed onset neurocognitive impairment (severe dementia and death) was problematic at conventional WBRT doses [9]. Therefore, chemotherapy-only approach or reduced dose WBRT for consolidation have been evaluated to reduce neurotoxicity. Also, high-dose chemotherapy with autologous stem cell transplant (auto-SCT) have resulted in improvements in outcomes in patients who responded to induction treatment [10–12], and listed as an option for consolidation therapy in NCCN guideline. However, about 90% of PCNSL cases are diffuse large B-cell lymphoma (DLBCL); therefore all these data are mostly based on treatment outcome for patients with DLBCL and there are scarce data regarding outcomes of non-DLBCL histologic subtypes.

## RESULTS

A total of 4375 patients with stage IE PCNSL were identified. The median age of the patients was 64 years (range, 18-96). DLBCL was the most common histologic subtype (N=3,091), followed by follicular lymphoma (FL, N=83), peripheral T-cell lymphoma (PTCL, N=64), marginal zone lymphoma (MZL, N=63), Burkitt lymphoma (BL, N=27), small lymphocytic lymphoma (SLL, N=22) and Hodgkin lymphoma (HL, N=13). In 1,012 patients (23%) the histologic subtype was recorded as “other” or “not otherwise specified” (Table 1).

**Table 1:** Patient characteristics

		All	DLBCL	FL	MZL	^*^PTCL	SLL	BL	HL
N		4,375	3,091	83	63	64	22	27	13
Median age	(range)	64 (18-96)	65 (18-96)	63 (30-86)	58 (23-92)	61 (18-83)	65 (41-81)	61 (30-77)	63 (38-80)
Male		2,336 (53)	1,628 (53)	42 (51)	16 (25)	36 (56)	9 (41)	18 (67)	8 (62)
Ethnicity	White	2,930 (67)	2,127 (69)	55 (66)	39 (62)	39 (61)	16 (72)	13 (48)	8 (62)
	Black	350 (8)	181 (6)	5 (6)	9 (14)	8 (13)	1 (5)	2 (7)	1 (8)
	Hispanic	616 (14)	421 (14)	16 (19)	8 (13)	12 (19)	3 (14)	6 (22)	4 (31)
	Asian/Pacific	445 (10)	334 (11)	7 (8)	7 (11)	5 (8)	2 (9)	6 (22)	
	Others	34 (1)	11 (1)						
Treatment	None	635 (15)	341 (11)	4 (5)	7 (11)	5 (8)	4 (18)	0 (0)	1 (8)
	Surgery only	328 (8)	240 (8)	12 (15)	13 (21)	6 (9)	1 (5)	1 (4)	2 (15)
	Radiation only	819 (19)	483 (16)	25 (30)	22 (35)	12 (19)	7 (32)	4 (15)	6 (46)
	Chemotherapy only	1,754 (40)	1,392 (45)	15 (18)	12 (19)	28 (44)	5 (23)	17 (63)	3 (23)
	Chemo-radiation	838 (19)	634 (21)	27 (33)	9 (14)	13 (20)	5 (23)	5 (19)	1 (8)

Abbreviation: DLBCL, diffuse large B-cell lymphoma; FL, follicular lymphoma; MZL, marginal zone lymphoma; PTCL, peripheral T-cell lymphoma; SLL, small lymphocytic lymphoma; BL, Burkitt lymphoma; HL, Hodgkin lymphoma.

^*^PTCL includes peripheral T-cell lymphoma-not otherwise specified, angioimmuoblastic T-cell lymphoma, anaplastic large cell lymphoma.

**Figure 1:**
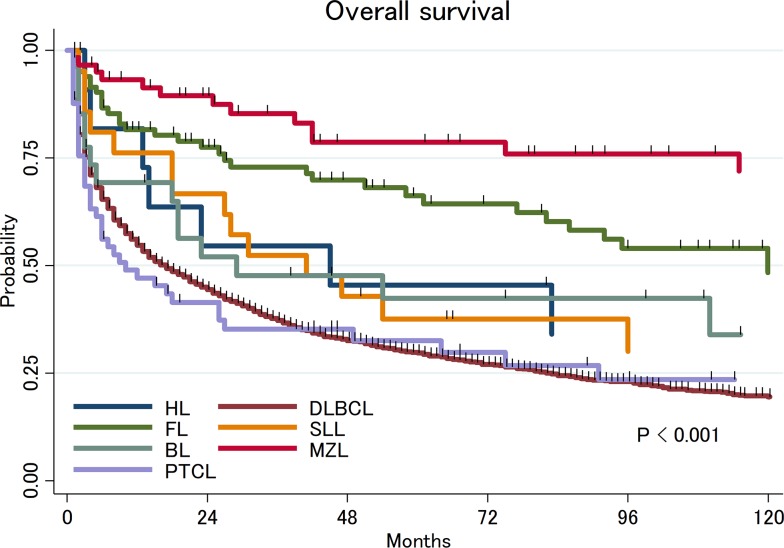
Overall survival of primary central nervous system lymphoma by histologic subtypes

Twenty-two percent of patients received neither radiation therapy (RT) nor chemotherapy, 19% of patients received RT alone, 40% of patients received chemotherapy alone and 19% of patients received combined modality therapy (CMT, chemo-radiation). Among patients who did not receive chemotherapy or radiation, 34% of patients underwent surgical resection. With a median follow up of 43 months (range: 1-202 months), 3,083 patients (70%) died and the median OS was 18 months (95%CI: 16-19). There was a significant difference in OS by histologic subtype (Figure 1): the 5-year OS rates were 30% (95%CI: 28-32%) in DLBCL, 66% (95%CI: 54-76%) in FL, 79% (95%CI: 64-88%) in MZL, 33% (95%CI: 20-45%) in PTCL, 42% (95%CI: 22-61%) in BL, 38% (95%CI: 18-57%) in SLL and 45% (95%CI: 17-71%) in HL. The differences in OS between each subtype are summarized in Supplementary Table 1. Multivariate analysis adjusted for age and treatment confirmed that patients with FL and MZL have significantly longer survival compared to patients with DLBCL (Table 2).

**Table 2:** Hazard ratio for OS compared to DLBCL

	HR	95%CI	P-value
DLBCL	Reference		
FL	0.32	0.23-0.46	<0.001
MZL	0.15	0.09-0.27	<0.001
PTCL	1.30	0.96-1.79	0.090
SLL	0.61	0.36-1.03	0.062
BL	0.86	0.52-1.40	0.540
HL	0.56	0.28-1.12	0.103

Abbreviation: HR, hazard ratio; CI, confidence interval; DLBCL, diffuse large B-cell lymphoma; FL, follicular lymphoma; MZL, marginal zone lymphoma; PTCL, peripheral T-cell lymphoma; SLL, small lymphocytic lymphoma; BL, Burkitt lymphoma; HL, Hodgkin lymphoma.

**Figure 2:**
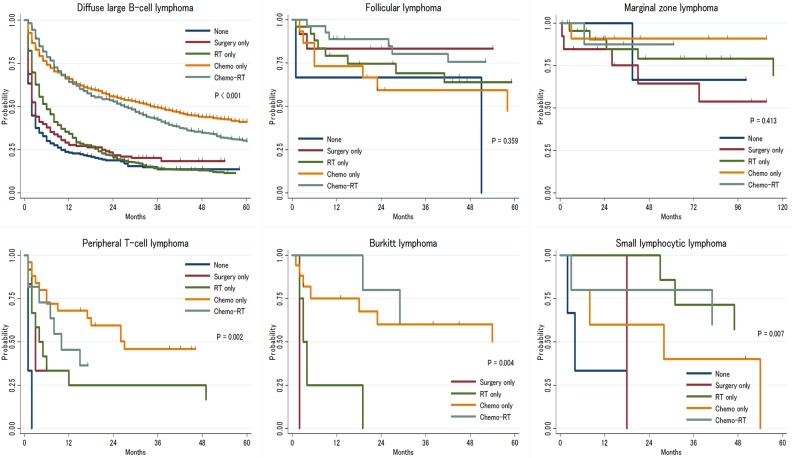
Overall survival of primary central nervous system lymphoma by treatment strategies

Response to therapy varied by histologic subtype. Among patients with aggressive histologic subtypes (DLBCL, BL and PTCL), radiation monotherapy was not associated with improvement in OS compared to no treatment. By contrast, among patients with indolent lymphomas (FL, MZL and SLL) radiation alone or resection without further therapy was associated with similar survival to chemotherapy or CMT (Figure 2).

**Figure 3:**
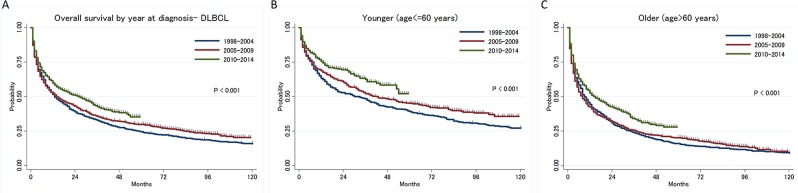
**(A)** Trend of overall survival in diffuse large B-cell lymphoma type primary central nervous system lymphoma by year at diagnosis, **(B)** in younger patients, **(C)** in older patients.

Among patients with DLBCL, there was a significant improvement in survival by treatment era. A survival benefit was seen in both younger (age≤60 years) and older (age>60 years) patients (Figure 3A–3C). There was no significant improvement in OS between 1998-2004 (N=853) and 2005-2009 (N=1,047) (median OS: 13 vs 14 months, p-value by log-rank test; all patients: P=0.075, younger: P=0.052, older: P=0.643) but there was a significant improvement in OS between 2005-2009 and 2010-2014 (N=1,191) (median OS: 14 vs 26 months, p-value by log-rank test; all patients: P<0.001, younger: P=0.018, older: P<0.001).

## DISCUSSION

PCNSL is a rare disease and there have been limited data available regarding survival outcomes of non-DLBCL PCNSL. Using registry data, we were able to analyze the incidence, treatment patterns and outcome of rare subtypes of PCNSL with a relatively large number of cases. Longer survival was seen in patients with MZL and FL compared to DLBCL.

In our study, the outcomes of patients with primary CNS T-cell lymphoma (TPCNSL) were comparable to DLBCL. In a multicenter international collaboration, Sjenkier *et al* described the clinical characteristics of 45 patients with TPCNSL [13]. The most common site of involvement was brain parenchyma (93%), and 19% of all patients also had cerebrospinal fluid involvement. With a median follow up of 22 months, the median progression-free survival and OS were 22 and 25 months respectively. This seems favorable compared to patients in our population-based cohort (median OS: 10 months). Unsurprisingly, the best outcomes were observed in patients with good performance status treated with MTX (median disease-specific survival 43 months). Treatment without HD-MTX was associated with inferior prognosis. MZL PCNSL was associated with the most favorable outcomes in our study. Recently, Ayanambakkam and colleagues systemically aggregated data from 69 patients with MZL PCNSL published as part of case series [14]. There was a female predominance (77%), concordant with our cohort (75%). Radiation ± surgery was the most common therapeutic approach (N=36), followed by CMT (N=16), surgery alone (N=11), while only 7 patients received chemotherapy ± surgery. After a median follow up of 23 months, only three relapses occurred with one death from sepsis, highlighting the indolent nature of disease.

Intensive approaches such as HD-MTX and auto-SCT have been shown to improve survival of patients with PCNSL; however, these approaches are only applicable to young and fit patients. Although our data showed improvement in survival outcomes for both younger and elderly patients, Mendez and colleagues recently showed that survival outcome of PCNSL is improving in young patients but not in elderly patients [15]. At the annual meeting of American Society of Hematology in 2016, multiple studies showed promising results by non-chemotherapy approaches, such as ibrutinib and lenalidomide, in PCNSL [16–18]. In the phase I study evaluating single agent ibrutinib for PCNSL, ORR was seen in 77% of patients (10 of 13 patients) including 38% if CR (5 of 13 patients) [19]. The efficacy of these drugs are in line with the specific biology of disease [20, 21], and they are relatively well tolerated in elderly patients. However, new drugs can be associated with new toxicities, and thus requires careful investigation. In the study evaluated ibrutinib combined with chemotherapy, increased aspergillosis was observed in patients [22]. Further studies are warranted to evaluate the role of these agents in PCNSL.

Several limitations should be noted. As this study uses cancer registry data, there is no review of pathology and therefore the accuracy of diagnosis is unknown. In a prior study, Clarke et al showed the accuracy of diagnoses particularly in rare subtypes in SEER registry data is suboptimal [23]. However, in this study we evaluated lymphoma subtypes encountered relatively frequently by pathologists (at least in their nodal presentation). Further, SEER data does not contain detailed treatment information, thus we were unable to evaluate the impact of induction chemotherapy, salvage regimens and auto-SCT. Our reported survival outcomes according to treatment modality should be interpreted in view of these limitations.

In conclusion, histologic subtype is a major determinant of prognosis among patients with PCNSL. Patients with indolent subtypes such as FL and MZL have favorable outcomes irrespective of treatment modality. The prognosis of patients with PCNSL-DLBCL remains inferior to patients with nodal DLBCL. Novel therapies such as ibrutinib and lenalidomide appear both active and less toxic. These agents may have an important role to play, considering the incidence of PCNSL is increasing in elderly patients.

## PATIENTS AND METHODS

The Surveillance, Epidemiology and End Results (SEER) 18 database, which currently covers approximately 27.8% of the US population, was used to evaluate overall survival (OS) of patients with PCNSL by different histologic subtypes in adult (≥18 years) patients diagnosed between 1998 and 2014. We only included stage I disease in this analysis but excluded primary vitreoretinal lymphoma. Overall survival (OS) was calculated from diagnosis to death from any cause using the Kaplan-Meier method. Differences in the survival function in different groups were analyzed using the log-rank test. Cox proportional hazards models were used to evaluate associations between patient characteristics and survival. All analyses were performed using STATA version 13.1 (StataCorp LP, College Station, TX), with significance set at the 5% level.

## SUPPLEMENTARY MATERIALS TABLE



## References

[1] Grommes C, DeAngelis LM (2017). Primary CNS Lymphoma. J Clin Oncol.

[2] Shiels MS, Pfeiffer RM, Besson C, Clarke CA, Morton LM, Nogueira L, Pawlish K, Yanik EL, Suneja G, Engels EA (2016). Trends in primary central nervous system lymphoma incidence and survival in the U.S. Br J Haematol.

[3] Network NCC (2017). Central Nervous System Cancers.

[4] Poortmans PM, Kluin-Nelemans HC, Haaxma-Reiche H, Van't Veer M, Hansen M, Soubeyran P, Taphoorn M, Thomas J, Van den Bent M, Fickers M, Van Imhoff G, Rozewicz C, Teodorovic I (2003). High-dose methotrexate-based chemotherapy followed by consolidating radiotherapy in non-AIDS-related primary central nervous system lymphoma: European Organization for Research and Treatment of Cancer Lymphoma Group Phase II Trial 20962. J Clin Oncol.

[5] DeAngelis LM, Seiferheld W, Schold SC, Fisher B, Schultz CJ, Radiation Therapy Oncology Group Study 93-10 (2002). Combination chemotherapy and radiotherapy for primary central nervous system lymphoma: Radiation Therapy Oncology Group Study 93-10. J Clin Oncol.

[6] Ferreri AJ, Reni M, Dell'Oro S, Ciceri F, Bernardi M, Camba L, Ponzoni M, Terreni MR, Tomirotti M, Spina M, Villa E (2001). Combined treatment with high-dose methotrexate, vincristine and procarbazine, without intrathecal chemotherapy, followed by consolidation radiotherapy for primary central nervous system lymphoma in immunocompetent patients. Oncology.

[7] O'Brien P, Roos D, Pratt G, Liew K, Barton M, Poulsen M, Olver I, Trotter G (2000). Phase II multicenter study of brief single-agent methotrexate followed by irradiation in primary CNS lymphoma. J Clin Oncol.

[8] DeAngelis LM, Yahalom J, Thaler HT, Kher U (1992). Combined modality therapy for primary CNS lymphoma. J Clin Oncol.

[9] Gavrilovic IT, Hormigo A, Yahalom J, DeAngelis LM, Abrey LE (2006). Long-term follow-up of high-dose methotrexate-based therapy with and without whole brain irradiation for newly diagnosed primary CNS lymphoma. J Clin Oncol.

[10] Shah GD, Yahalom J, Correa DD, Lai RK, Raizer JJ, Schiff D, LaRocca R, Grant B, DeAngelis LM, Abrey LE (2007). Combined immunochemotherapy with reduced whole-brain radiotherapy for newly diagnosed primary CNS lymphoma. J Clin Oncol.

[11] Omuro A, Correa DD, DeAngelis LM, Moskowitz CH, Matasar MJ, Kaley TJ, Gavrilovic IT, Nolan C, Pentsova E, Grommes CC, Panageas KS, Baser RE, Faivre G (2015). R-MPV followed by high-dose chemotherapy with TBC and autologous stem-cell transplant for newly diagnosed primary CNS lymphoma. Blood.

[12] Ferreri AJ, Cwynarski K, Pulczynski E, Ponzoni M, Deckert M, Politi LS, Torri V, Fox CP, Rosee PL, Schorb E, Ambrosetti A, Roth A, Hemmaway C (2016). Chemoimmunotherapy with methotrexate, cytarabine, thiotepa, and rituximab (MATRix regimen) in patients with primary CNS lymphoma: results of the first randomisation of the International Extranodal Lymphoma Study Group-32 (IELSG32) phase 2 trial. Lancet Haematol.

[13] Shenkier TN, Blay JY, O'Neill BP, Poortmans P, Thiel E, Jahnke K, Abrey LE, Neuwelt E, Tsang R, Batchelor T, Harris N, Ferreri AJ, Ponzoni M (2005). Primary CNS lymphoma of T-cell origin: a descriptive analysis from the international primary CNS lymphoma collaborative group. J Clin Oncol.

[14] Ayanambakkam A, Ibrahimi S, Bilal K, Cherry MA (2018). Extranodal marginal zone lymphoma of the central nervous system. Clin Lymphoma Myeloma Leuk.

[15] Mendez JS, Ostrom QT, Gittleman H, Kruchko C, DeAngelis LM, Barnholtz-Sloan JS, Grommes C (2018). The elderly left behind - changes in survival trends of primary central nervous system lymphoma over the past four decades. Neuro Oncol.

[16] Grommes C, Pastore A, Gavrilovic I, Kaley T, Nolan C, Omuro AM, Wolfe J, Pentsova E, Hatzoglou V, Mellinghoff I, DeAngelis L (2016). Single-agent ibrutinib in recurrent/refractory central nervous system lymphoma. Blood.

[17] Ghesquieres H, Houillier C, Chinot O, Choquet S, Molucon-Chabrot C, Beauchene P, Gressin R, Morschhauser F, Schmitt A, Gyan E, Hoang-Xuan K, Nicolas-Virelizier E, Chevrier M (2016). Rituximab-Lenalidomide (REVRI) in Relapse or Refractory Primary Central Nervous System (PCNSL) or Vitreo Retinal Lymphoma (PVRL): Results of a “Proof of Concept” Phase II Study of the French LOC Network. Blood.

[18] Choquet S, Houillier C, Bijou F, Houot R, Boyle E, Gressin R, Nicolas-Virelizier E, Barrie M, Molucon-Chabrot C, Blonski M, El Yamani A, LeLez ML, Clavert A (2016). Ibrutinib monotherapy in relapse or refractory primary CNS lymphoma (PCNSL) and Primary Vitreo-Retinal Lymphoma (PVRL). Result of the Interim Analysis of the iLOC Phase II Study from the Lysa and the French LOC Network. Blood.

[19] Grommes C, Pastore A, Palaskas N, Tang SS, Campos C, Schartz D, Codega P, Nichol D, Clark O, Hsieh WY, Rohle D, Rosenblum M, Viale A (2017). Ibrutinib Unmasks Critical Role of Bruton Tyrosine Kinase in Primary CNS Lymphoma. Cancer Discov.

[20] Chapuy B, Roemer MG, Stewart C, Tan Y, Abo RP, Zhang L, Dunford AJ, Meredith DM, Thorner AR, Jordanova ES, Liu G, Feuerhake F, Ducar MD (2016). Targetable genetic features of primary testicular and primary central nervous system lymphomas. Blood.

[21] Gonzalez-Aguilar A, Idbaih A, Boisselier B, Habbita N, Rossetto M, Laurenge A, Bruno A, Jouvet A, Polivka M, Adam C, Figarella-Branger D, Miquel C, Vital A (2012). Recurrent mutations of MYD88 and TBL1XR1 in primary central nervous system lymphomas. Clin Cancer Res.

[22] Lionakis MS, Dunleavy K, Roschewski M, Widemann BC, Butman JA, Schmitz R, Yang Y, Cole DE, Melani C, Higham CS, Desai JV, Ceribelli M, Chen L (2017). Inhibition of B Cell Receptor Signaling by Ibrutinib in Primary CNS Lymphoma. Cancer Cell.

[23] Clarke CA, Undurraga DM, Harasty PJ, Glaser SL, Morton LM, Holly EA (2006). Changes in cancer registry coding for lymphoma subtypes: reliability over time and relevance for surveillance and study. Cancer Epidemiol Biomarkers Prev.

