# Supplementary material for: Impact of histologic subtypes and treatment modality among patients with primary central nervous system lymphoma: a SEER database analysis
Source: Oncotarget. 2018 Jun 22;9(48):28897–902. doi: 10.18632/oncotarget.25622 (PMC6034756; doi:10.18632/oncotarget.25622)
Supplement: Supplementary file 1 [file oncotarget-09-28897-s001.pdf]

Impact of histologic subtypes and treatment modality among patients with primary central nervous system lymphoma: a SEER database analysis

SUPPLEMENTARY MATERIALS

Supplementary Table 1: Logrank test between each subtype

	HL	DLBCL	FL	SLL	BL	MZL	PTCL
HL		0.243	0.120	0.882	0.947	0.002	0.331
DLBCL			<0.001	0.109	0.153	<0.001	0.800
FL				0.072	0.027	0.020	<0.001
SLL					0.822	0.001	0.135
BL						<0.001	0.231
MZL							<0.001
PTCL							
